# Supplementary material for: Major discrepancy between factual antibiotic resistance and consumption in South of France: analysis of 539,037 bacterial strains
Source: Sci Rep. 2020 Oct 26;10:18262. doi: 10.1038/s41598-020-75158-7 (PMC7588456; doi:10.1038/s41598-020-75158-7)
Supplement: Supplementary file 1 — Supplementary information. [file 41598_2020_75158_MOESM1_ESM.pdf]

Full-length title: Major discrepancy between factual antibiotic resistance and consumption in South of France: analysis of 539,037 bacterial strains.

Author list: Ousmane Oumou Diallo[1,2], Sophie Alexandra Baron[1,2], Gregory Dubourg[1,2], Hervé Chaudet[2,3], Philippe Halfon[4], Sabine Camiade[4], Béatrice Comte[5], Stéphanie Joubert[5], Arnaud François[5], Philippe Seyral[6], François Parisot[6], Jean-Paul Casalta[3,7], Raymond Ruimy[8], Christophe Maruejouls[9], Jean-Christophe Achiardy[9], Sophie Burignat[10], Joseph Carvajal[10], Edouard Delaunay[10], Sandra Meyer[10], Pierre-Yves Levy[3,11], Patricia Roussellier[12], Patrick Brunet[13], Claude Bosi[14], Philippe Stolidi[14], Jean-Pierre Arzouni[3,15], Gisele Gay[15], Pierre Hance[15], Philippe Colson[1,2] , Didier Raoult[1,2*] and Jean-Marc Rolain[1,2*]

Affiliations: [1] Aix Marseille Univ, IRD, APHM, MEPHI, Marseille, France, [2] MEPHI, Aix Marseille Université, IHU Méditerranée Infection, AP-HM, Marseille, [3] VITROME, Aix Marseille Université, IHU Méditerranée Infection, AP-HM, Marseille, [4]LBM Alphabio Marseille, [5]LBM Bioesterel, Cannes, [6]LBM Labazur Nice, [7]LBM Labazur Provence, [8]Laboratoire de bactériologie, CHU Nice, [9]LBM Barla, Nice, [10]LBM Cerballiance, Marseille, [11]LBM, Casamance, Aubagne, [12]Laboratoire de microbiologie, CH, Salon de Provence, [13]Laboratoire de microbiologie, CH Saint-Joseph, Marseille, [14]Laboratoire de microbiologie, CH, Aubagne, [15]LBM, Labosud Provence Biologie, Marseille

* **Corresponding author :** Jean-Marc Rolain, Email: jean-marc.rolain@univ-amu.fr. Didier Raoult, Email: didier.raoult@gmail.com. Aix Marseille Univ, IRD, APHM, MEPHI, IHU Méditerranée Infection, Faculté de Médecine et de Pharmacie, 19-21 boulevard Jean Moulin, 13385 Marseille CEDEX 05, France. Phone: (33) 4 91 32 43 75.

Table S1. Presentation of the different bacterial species isolated from our surveillance

Bacterial species	Number of strains
Escherichia coli	284242
Staphylococcus aureus	69070
Klebsiella pneumoniae	54719
Enterococcus faecalis	41158
Pseudomonas aeruginosa	36911
Staphylococcus epidermidis	29663
Proteus mirabilis	20510
Enterobacter cloacae	18477
Streptococcus agalactiae	15809
Citrobacter koseri	8658
Klebsiella oxytoca	8650
Enterobacter aerogenes	7689
Haemophilus influenzae	7456
Staphylococcus saprophyticus	5512
Morganella morganii	5386
Staphylococcus hominis	5312
Enterococcus faecium	5089
Staphylococcus haemolyticus	4757
Serratia marcescens	4684
Streptococcus pyogenes	4078
Citrobacter freundii	4022
Stenotrophomonas maltophilia	3395
Staphylococcus lugdunensis	3171
Streptococcus pneumoniae	2888
Staphylococcus capitis	2757
Propionibacterium acnes	2515
Streptococcus anginosus	2232
Streptococcus constellatus	1875
Corynebacterium striatum	1847
Moraxella catarrhalis	1839
Campylobacter jejuni	1249
Proteus vulgaris	1207
Bacteroides fragilis	1198
Aerococcus urinae	1197
Achromobacter xylosoxidans	1181
Klebsiella variicola	1179
Haemophilus parainfluenzae	1171
Streptococcus mitis	1165
Streptococcus dysgalactiae	1153
Acinetobacter baumannii	1123
Streptococcus oralis	1090
Salmonella sp	1087

Hafnia alvei	947
Streptococcus gallolyticus	776
Acinetobacter pittii	764
Staphylococcus warneri	673
Staphylococcus sp	657
Raoultella ornithinolytica	629
Streptococcus sp	600
Pseudomonas putida	561
Micrococcus luteus	551
Providencia stuartii	545
Streptococcus intermedius	497
Corynebacterium amycolatum	438
Neisseria gonorrhoeae	427
Cutibacterium acnes	413
Providencia rettgeri	402
Pasteurella multocida	399
Enterococcus avium	391
Clostridium difficile	384
Staphylococcus simulans	382
Finegoldia magna	378
Haemophilus haemolyticus	367
Citrobacter braakii	358
Bacillus cereus	342
Staphylococcus caprae	341
Corynebacterium sp	335
Acinetobacter baumanii	327
Streptococcus salivarius	321
Turicella otitidis	310
Enterobacter kobei	308
Campylobacter coli	278
Pseudomonas sp	278
Propionibacterium avidum	271
Actinotignum schaalii	267
Enterobacter sp	264
Enterobacter asburiae	253
Aeromonas hydrophila	250
Corynebacterium glucuronolyticum	250
Corynebacterium urealyticum	235
Parvimonas micra	231
Streptococcus parasanguinis	222
Bacteroides thetaiotaomicron	220
Haemophilus parahaemolyticus	212
Staphylococcus pasteuri	212
Corynebacterium tuberculostearicum	204

Actinomyces odontolyticus	203
Corynebacterium jeikeium	189
Actinomyces neuii	187
Enterococcus gallinarum	183
Streptococcus sanguinis	182
Acinetobacter ursingii	172
Actinomyces turicensis	172
Staphylococcus pettenkoferi	172
Lactobacillus gasseri	170
Corynebacterium pseudodiphtheriticum	169
Dermabacter hominis	166
Clostridium perfringens	162
Aeromonas caviae	160
Citrobacter amalonaticus	156
Peptoniphilus harei	154
Corynebacterium propinquum	153
Aeromonas sp	152
Capnocytophaga sputigena	152
Klebsiella aerogenes	152
Lactobacillus rhamnosus	151
Acinetobacter sp	147
Enterococcus durans	145
Lactobacillus delbrueckii	143
Prevotella bivia	143
Neisseria meningitidis	140
Enterococcus casseliflavus	137
Proteus hauseri	129
Acinetobacter junii	127
Yersinia enterocolitica	127
Eikenella corrodens	125
Staphylococcus cohnii	125
Staphylococcus pseudintermedius	125
Citrobacter werkmanii	123
Lactobacillus jensenii	123
Pantoea agglomerans	123
Corynebacterium simulans	120
Lactobacillus iners	120
Fusobacterium nucleatum	115
Corynebacterium accolens	114
Streptococcus gordonii	112
Staphylococcus schleiferi	111
Acinetobacter lwoffii	110
Moraxella osloensis	110
Serratia liquefaciens	108

Corynebacterium aurimucosum	102
Actinomyces sp	100
Citrobacter sp	95
Bacteroides vulgatus	94
Enterococcus sp	94
Rothia mucilaginosa	93
Pantoea sp	92
Aerococcus sanguinicola	91
Salmonella enterica	91
Enterococcus hirae	90
Bacillus sp	89
Aeromonas veronii	86
Eggerthella lenta	84
Pasteurella canis	83
Pseudomonas oryzihabitans	83
Burkholderia multivorans	80
Proteus penneri	80
Moraxella nonliquefaciens	79
Campylobacter fetus	77
Achromobacter sp	76
Alcaligenes faecalis	75
Staphylococcus intermedius	75
Bacteroides ovatus	74
Raoultella planticola	74
Delftia acidovorans	72
Corynebacterium macginleyi	69
Helicobacter pylori	69
Streptococcus vestibularis	69
Veillonella parvula	68
Enterobacter ludwigii	67
Pseudomonas stutzeri	67
Actinomyces oris	66
Cutibacterium avidum	66
Neisseria flavescens	62
Pseudomonas mosselii	62
Shigella sonnei	61
Aerococcus viridans	60
Acinetobacter nosocomialis	59
Citrobacter farmeri	59
Propionibacterium	59
Aggregatibacter aphrophilus	57
Neisseria sp	56
Moraxella sp	54
Staphylococcus saccharolyticus	53

Neisseria macacae	52
Clostridium tertium	51
Enterobacter cancerogenus	51
Fusobacterium necrophorum	51
Kocuria rhizophila	50
Streptococcus lutetiensis	50
Streptococcus pasteurianus	50
Bacillus pumilus	49
Enterobacter hormaechei	49
Citrobacter youngae	48
Acinetobacter johnsonii	47
Burkholderia cenocepacia	47
Aggregatibacter segnis	46
Leclercia adecarboxylata	46
Propionibacterium granulosum	46
Staphylococcus auricularis	46
Pseudomonas monteilii	45
Bacteroides uniformis	44
Peptostreptococcus anaerobius	43
Brevibacterium casei	42
Peptoniphilus asaccharolyticus	42
Streptococcus canis	42
Acinetobacter radioresistens	41
Pandoraea pulmonicola	41
Aeromonas sobria	40
Escherichia hermannii	40
Granulicatella adiacens	40
Citrobacter sedlakii	39
Corynebacterium coyleae	39
Prevotella buccae	39
Lactobacillus sp	38
Actinomyces neuii ssp neuii	37
Actinomyces urogenitalis	37
Lactococcus lactis	37
Streptococcus massiliensis	37
Klebsiella sp	36
Listeria monocytogenes	36
Roseomonas mucosa	36
Trueperella bernardiae	36
Haemophilus sp	35
Ochrobactrum anthropi	34
Parabacteroides distasonis	34
Pseudomonas fluorescens	34
Actinomyces radingae	33

Lactobacillus casei	32
Actinomyces europaeus	31
Sphingomonas paucimobilis	31
Streptococcus mutans	31
Aerococcus sp	30
Enterococcus raffinosus	30
Gemella morbillorum	30
Shigella sp	30
Streptococcus pseudopneumoniae	30
Achromobacter denitrificans	29
Bacteroides sp	29
Erysipelatoclostridium ramosum	29
Nocardia sp	29
Achromobacter insolitus	28
Prevotella denticola	28
Pseudomonas mendocina	28
Abiotrophia defectiva	27
Clostridium clostridioforme	27
Corynebacterium minutissimum	27
Moraxella lacunata	27
Proteus sp	27
Streptococcus australis	27
Chryseobacterium gleum	26
Rhizobium radiobacter	26
Rothia dentocariosa	26
Staphylococcus petrasii	26
Acinetobacter haemolyticus	25
Bacteroides pyogenes	25
Kocuria sp	25
Lactobacillus paracasei	25
Prevotella intermedia	25
Staphylococcus xylosus	25
Agrobacterium tumefaciens	24
Gemella haemolysans	24
Streptococcus equinus	24
Bordetella bronchiseptica	23
Pantoea septica	23
Acinetobacter septicus	22
Arcanobacterium haemolyticum	22
Bacteroides caccae	22
Chryseobacterium indologenes	22
Pseudomonas luteola	22
Staphylococcus condimenti	22
Streptococcus cristatus	22

Burkholderia cepacia	21
Cronobacter sakazakii	21
Escherichia vulneris	21
Prevotella sp	21
Pseudomonas fulva	21
Lactobacillus fermentum	20
Prevotella melaninogenica	20
Acinetobacter calcoaceticus	19
Clostridium ramosum	19
Clostridium septicum	19
Corynebacterium afermentans	19
Elizabethkingia meningoseptica	19
Fusobacterium sp	19
Pasteurella sp	19
Anaerococcus vaginalis	18
Paenibacillus sp	18
Pasteurella stomatis	18
Pseudoglutamicibacter cumminsii	18
Serratia rubidaea	18
Streptococcus thermophilus	18
Alloscardovia omnicolens	17
Brevundimonas diminuta	17
Capnocytophaga sp	17
Dolosigranulum pigrum	17
Kocuria kristinae	17
Microbacterium sp	17
Oligella urethralis	17
Pseudomonas plecoglossicida	17
Serratia sp	17
Shigella flexneri	17
Bacillus subtilis	16
Campylobacter sp	16
Lactococcus garvieae	16
Pantoea dispersa	16
Pasteurella dagmatis	16
Raoultella sp	16
Shewanella putrefaciens	16
Streptococcus infantarius	16
Arthrobacter sp	15
Bacillus simplex	15
Bifidobacterium breve	15
Burkholderia gladioli	15
Clostridium innocuum	15
Dialister pneumosintes	15

Kluyvera ascorbata	15
Lactobacillus salivarius	15
Neisseria subflava	15
Peptoniphilus sp	15
Staphylococcus sciuri	15
Atopobium parvulum	14
Haemophilus sputorum	14
Lactobacillus johnsonii	14
Vibrio alginolyticus	14
Actinomyces naeslundii	13
Fusobacterium naviforme	13
Pantoea calida	13
Prevotella disiens	13
Veillonella atypica	13
Fusobacterium gonidiaformans	12
Kingella kingae	12
Lelliottia amnigena	12
Prevotella oris	12
Salmonella typhimurium	12
Serratia ureilytica	12
Actinomyces meyeri	11
Arcobacter butzleri	11
Bacillus megaterium	11
Burkholderia sp	11
Clostridium sp	11
Corynebacterium bovis	11
Pandoraea sputorum	11
Roseomonas sp	11
Staphylococcus equorum	11
Acinetobacter bereziniae	10
Anaerococcus octavius	10
Bacillus circulans	10
Bacillus thuringiensis	10
Bacteroides faecis	10
Chryseobacterium sp	10
Clostridium sordellii	10
Cutibacterium granulosum	10
Helcococcus kunzii	10
Lactobacillus crispatus	10
Ochrobactrum intermedium	10
Paracoccus yeei	10
Actinomyces ihumii	9
Actinomyces viscosus	9
Actinotignum sanguinis	9

Bacillus licheniformis	9
Clostridium sphenoides	9
Clostridium sporogenes	9
Corynebacterium imitans	9
Corynebacterium kroppenstedtii	9
Pseudomonas alcaligenes	9
Serratia odorifera	9
Staphylococcus carnosus	9
Streptococcus equisimilis	9
Acinetobacter schindleri	8
Aggregatibacter sp	8
Bifidobacterium sp	8
Bordetella spp	8
Clostridium paraputrificum	8
Comamonas testosteroni	8
Corynebacterium argentoratense	8
Corynebacterium mucifaciens	8
Ewingella americana	8
Haemophilus pittmaniae	8
Kluyvera cryocrescens	8
Nocardia farcinica	8
Ochrobactrum sp	8
Peptoniphilus gorbachii	8
Pluralibacter gergoviae	8
Prevotella nigrescens	8
Pseudomonas koreensis	8
Rothia sp	8
Serratia ficaria	8
Sphingomonas sp	8
Veillonella sp	8
Vibrio parahaemolyticus	8
Atopobium vaginae	7
Bifidobacterium longum	7
Brevibacterium sp	7
Corynebacterium diphtheriae	7
Fusobacterium varium	7
Gemella sp	7
Lactobacillus acidophilus	7
Leptotrichia buccalis	7
Microbacterium paraoxydans	7
Neisseria mucosa	7
Porphyromonas somerae	7
Raoultella terrigena	7
Ruminococcus gnavus	7

Serratia fonticola	7
Stenotrophomonas sp	7
Actinobaculum massiliense	6
Arcobacter cryaerophilus	6
Capnocytophaga gingivalis	6
Corynebacterium auris	6
Corynebacterium confusum	6
Lactobacillus plantarum	6
Leuconostoc lactis	6
Neisseria weaveri	6
Nocardia abscessus	6
Pasteurella bettyae	6
Peptostreptococcus sp	6
Plesiomonas shigelloides	6
Prevotella baroniae	6
Prevotella loescheii	6
Pseudomonas otitidis	6
Rahnella aquatilis	6
Salmonella enteritidis	6
Solobacterium moorei	6
Streptococcus equi	6
Streptococcus porcinus	6
Streptococcus zooepidemicus	6
Acinetobacter guillouiae	5
Actinobaculum sp	5
Actinomyces israelii	5
Actinomyces turicencis	5
Anaerococcus sp	5
Bacteroides cellulosilyticus	5
Bifidobacterium bifidum	5
Capnocytophaga ochracea	5
Comamonas kerstersii	5
Corynebacterium pseudotuberculosis	5
Delftia sp	5
Elizabethkingia miricola	5
Facklamia hominis	5
Kocuria varians	5
Leuconostoc mesenteroides	5
Listeria sp	5
Lysinibacillus fusiformis	5
Prevotella oralis	5
Rhizobium sp	5
Roseomonas gilardii	5
Serratia proteamaculans	5

Shewanella algae	5
Shigella boydii	5
Slackia exigua	5
Staphylococcus piscifermentans	5
Acinetobacter parvus	4
Actinobacillus sp	4
Actinomyces graevenitzii	4
Actinotignum sp	4
Aggregatibacter actinomycetemcomitans	4
Atopobium rimae	4
Bacillus mycoides	4
Bacteroides stercoris	4
Bifidobacterium scardovii	4
Branhamella sp	4
Brevibacterium celere	4
Brevibacterium luteolum	4
Brevibacterium paucivorans	4
Comamonas sp	4
Corynebacterium ihumii	4
Corynebacterium ulcerans	4
Delftia tsuruhatensis	4
Dialister micraerophilus	4
Empedobacter brevis	4
Empedobacter falsenii	4
Erysipelothrix rhusiopathiae	4
Gemella sanguinis	4
Granulicatella elegans	4
Hungatella hathewayi	4
Kocuria palustris	4
Kytococcus schroeteri	4
Lactobacillus sakei	4
Leptotrichia sp	4
Leptotrichia trevisanii	4
Leuconostoc sp	4
Macrococcus caseolyticus	4
Micrococcus sp	4
Neisseria elongata	4
Pediococcus pentosaceus	4
Peptococcus sp	4
Prevotella nanceiensis	4
Propionibacterium sp	4
Serratia plymuthica	4
Shigella dysenteriae	4
Staphylococcus urealyticus	4

Streptococcus urinalis	4
Actinomyces grossensis	3
Anaerococcus murdochii	3
Bacillus vallismortis	3
Bergeyella zoohelcum	3
Bordetella petrii	3
Brevundimonas aurantiaca	3
Brevundimonas vesicularis	3
Brucella sp	3
Burkholderia pseudomallei	3
Campylobacter upsaliensis	3
Clostridium bifermentans	3
Clostridium subterminale	3
Corynebacterium lascolaensis	3
Corynebacterium singulare	3
Corynebacterium ureicelerivorans	3
Cupriavidus respiraculi	3
Dermabacter sp	3
Eggerthella sp	3
Enterococcus malodoratus	3
Escherichia fergusonii	3
Facklamia languida	3
Fusobacterium mortiferum	3
Granulicatella sp	3
Inquilinus limosus	3
Kluyvera intermedia	3
Kosakonia cowanii	3
Microbacterium oxydans	3
Micrococcus lylae	3
Myroides sp	3
Neisseria lactamica	3
Nocardia cyriacigeorgica	3
Parabacteroides goldsteinii	3
Prevotella conceptionensis	3
Prevotella corporis	3
Providencia alcalifaciens	3
Providencia sp	3
Pseudomonas massiliensis	3
Staphylococcus gallinarum	3
Streptococcus minor	3
Sutterella sp	3
Vagococcus fluvialis	3
Weeksella virosa	3
Weissella confusa	3

Weissella viridescens	3
Yersinia sp	3
Achromobacter ruhlandii	2
Aeromonas jandaei	2
Aeromonas media	2
Alistipes finegoldii	2
Anaerococcus lactolyticus	2
Anaerococcus prevotii	2
Anaerococcus tetradius	2
Arcanobacterium sp	2
Arcobacter sp	2
Aureimonas altamirensis	2
Bacillus altitudinis	2
Bacillus amyloliquefaciens	2
Bacillus vietnamensis	2
Bacillus weihenstephanensis	2
Bacteroides heparinolyticus	2
Bifidobacterium dentium	2
Bordetella hinzii	2
Brevibacterium ravenspurgense	2
Brevundimonas sp	2
Budvicia aquatica	2
Cellulosimicrobium cellulans	2
Clostridium baratii	2
Collinsella aerofaciens	2
Coprobacillus sp	2
Corynebacterium freneyi	2
Corynebacterium riegelii	2
Corynebacterium xerosis	2
Cupriavidus gilardii	2
Eikenella sp	2
Enterobacter amnigenus	2
Erwinia sp	2
Escherichia sp	2
Exiguobacterium	2
Flavobacterium sp	2
Flavonifractor plautii	2
Gordona bronchialis	2
Gordonia sp	2
Ignavigranum ruoffiae	2
Janibacter hoylei	2
Kerstersia gyiorum	2
Kluyvera georgiana	2
Lactococcus sp	2

Leuconostoc citreum	2
Listeria ivanovii	2
Microbacterium kitamiense	2
Neisseria animaloris	2
Neisseria canis	2
Neisseria cinerea	2
Neisseria flava	2
Nocardia nova	2
Paracoccus sp	2
Pediococcus sp	2
Peptoniphilus grossensis	2
Porphyromonas asaccharolytica	2
Porphyromonas gingivalis	2
Prevotella buccalis	2
Pseudomonas citronellolis	2
Pseudomonas oleovorans	2
Pseudopropionibacterium propionicum	2
Ralstonia sp	2
Sphingobacterium multivorum	2
Staphylococcus felis	2
Staphylococcus kloosii	2
Streptococcus castoreus	2
Streptococcus infantis	2
Streptococcus peroris	2
Streptococcus uberis	2
Tissierella praeacuta	2
Veillonella dispar	2
Weissella sp	2
Yersinia pseudotuberculosis	2
Acidovorax temperans	1
Actinobacillus pleuropneumoniae	1
Actinomyces bowdenii	1
Actinomyces dentalis	1
Actinomyces georgiae	1
Actinomyces radicidentis	1
Aeromonas bestiarum	1
Aeromonas encheleia	1
Aeromonas salmonicida	1
Akkermansia muciniphila	1
Alcaligenes sp	1
Alloiococcus otitis	1
Anaerococcus hydrogenalis	1
Anaerotruncus colihominis	1
Bacillus badius	1

Bacillus clausii	1
Bacillus firmus	1
Bacillus horneckiae	1
Bacillus infantis	1
Bacillus marisflavi	1
Bacteroides massiliensis	1
Bacteroides nordii	1
Bacteroides salyersiae	1
Bergeyella sp	1
Bordetella trematum	1
Brevibacillus agri	1
Brevibacillus parabrevis	1
Brevibacterium massiliense	1
Brucella melitensis	1
Burkholderia stabilis	1
Butyricimonas phoceencis	1
Butyricimonas virosa	1
Campylobacter lari	1
Campylobacter rectus	1
Campylobacter sputorum	1
Capnocytophaga canimorsus	1
Capnocytophaga granulosa	1
Carnobacterium divergens	1
Carnobacterium maltaromaticum	1
Cellulomonas sp	1
Chryseobacterium hominis	1
Citrobacter gillenii	1
Citrobacter murliniae	1
Clostridium aldenense	1
Clostridium butyricum	1
Clostridium cadaveris	1
Clostridium celerecrescens	1
Clostridium tetani	1
Corynebacterium auriscanis	1
Corynebacterium durum	1
Corynebacterium glutamicum	1
Corynebacterium hansenii	1
Corynebacterium lipophile	1
Corynebacterium pilosum	1
Cupriavidus sp	1
Desulfovibrio desulfuricans	1
Dialister sp	1
Dietzia cinnamea	1
Dietzia natronolimnaea	1

Enterococcus devriesei	1
Enterococcus mundtii	1
Erwinia persicina	1
Eubacterium limosum	1
Fusobacterium canifelinum	1
Fusobacterium periodonticum	1
Gardnerella vaginalis	1
Gemella bergeri	1
Gordonia bronchialis	1
Haemophilus haemoglobinophilus	1
Haemophilus parasuis	1
Helcococcus sueciensis	1
Janibacter sanguinis	1
Kingella sp	1
Kluyvera sp	1
Kytococcus sedentarius	1
Lactobacillus mucosae	1
Lactobacillus murinus	1
Lactobacillus reuteri	1
Lactobacillus vaginalis	1
Lysinibacillus massiliensis	1
Massilia timonae	1
Microbacterium aurum	1
Microbacterium flavescens	1
Microbacterium lacticum	1
Micrococcus flavus	1
Moraxella atlantae	1
Moraxella lincolnii	1
Morganella sp	1
Mycobacterium abscessus	1
Mycobacterium avium	1
Mycobacterium chelonae	1
Mycobacterium sp	1
Myroides odoratimimus	1
Neisseria zoodegmatis	1
Nocardia wallacei	1
Olsenella sp	1
Paenibacillus amylolyticus	1
Paenibacillus durus	1
Paenibacillus lactis	1
Paenibacillus pabuli	1
Paenibacillus provencensis	1
Paenibacillus urinalis	1
Pantoea annanatis	1

Parabacteroides merdae	1
Pediococcus acidilactici	1
Peptoniphilus indolicus	1
Peptoniphilus olsenii	1
Peptoniphilus tyrrelliae	1
Peptostreptococcus stomatis	1
Photobacterium damselae	1
Porphyromonas sp	1
Prevotella bergensis	1
Prevotella dentalis	1
Prevotella massiliensis	1
Prevotella oulorum	1
Prevotella pallens	1
Prevotella timonensis	1
Prevotella veroralis	1
Propionibacterium propionicum	1
Propionimicrobium lymphophilum	1
Pseudomonas agarici	1
Pseudomonas kuykendallii	1
Pseudomonas libanensis	1
Pseudomonas pseudoalcaligenes	1
Pseudomonas synxantha	1
Rhodococcus equi	1
Rhodococcus erythropolis	1
Rhodococcus rhodochrous	1
Rhodococcus sp	1
Rodentibacter pneumotropicus	1
Roseomonas genomospecies	1
Rothia terrae	1
Serratia grimesii	1
Sphingobacterium sp	1
Sphingobacterium spiritivorum	1
Sphingopyxis sp	1
Sporolactobacillus laevolacticus	1
Sporosarcina luteola	1
Staphylococcus arlettae	1
Staphylococcus delphini	1
Staphylococcus succinus	1
Stenotrophomonas acidaminiphila	1
Stenotrophomonas rhizophila	1
Streptococcus alactolyticus	1
Streptococcus ovis	1
Streptococcus pseudoporcinus	1
Streptococcus sobrinus	1

Sutterella wadsworthensis	1
Terrisporobacter glycolicus	1
Turicibacter sanguinis	1
Vagococcus sp	1
Vibrio cholerae	1
Vibrio fluvialis	1
Vibrio vulnificus	1
Wautersiella falsenii	1
Yersinia frederiksenii	1
Yersinia intermedia	1
Yersinia kristensenii	1
Yokenella regensburgei	1
Total	711032

: system from January 2014 to February 2019 (N=711,031).

Table S2: Trend in antibiotic resistance of the species studied for the full years 2014 to 2018.

	2014			2015			2016			2017			2018		
Escherichia coli															
	R	N	%	R	N	%	R	N	%	R	N	%	R	N	%
CRO	2,046	21,067	9.71	2,882	25,808	11.17	5,026	50,968	9.86	6,554	74,218	8.83	7,551	84,657	8.92
AMX	13,380	22,770	58.76	15,651	27,344	57.24	25,510	52,005	49.05	37,087	76,370	48.56	41,266	85,446	48.29
AMC	9,052	22,794	39.71	9821	25,420	38.63	10,061	42,299	23.79	14,160	67,295	21.04	14,347	66,259	21.65
CIP	4,135	22,801	18.14	4,775	27,288	17.50	6,797	43,957	15.46	12,005	76,217	15.75	8,492	67,992	12.49
IPM	18	16,693	0.11	23	17,532	0.13	38	17,696	0.21	27	26,242	0.10	72	28,767	0.25
AN	792	16,733	4.73	540	21,303	2.53	1,743	46,367	3.76	1,791	70,734	2.53	1,105	80,977	1.36
Proteus mirabilis															
AMX	867	2,025	42.81	856	2,145	39.91	1,462	3,639	40.18	2,092	5,328	39.26	2,485	5,966	41.65
AMC	382	2,027	18.85	334	2,034	16.42	298	3,056	9.75	391	4,805	8.14	363	4,798	7.57
CRO	41	1,880	2.18	56	2,007	2.79	65	3,464	1.88	91	5,065	1.80	88	5,749	1.53
CIP	260	2,030	12.81	251	2,139	11.73	416	3,241	12.84	702	5,318	13.20	527	4,895	10.77
IPM	18	1,615	1.11	28	1,518	1.84	26	1,512	1.72	37	1,629	2.27	74	1,471	5.03

AN	76	1,390	5.47	34	1,595	2.13	106	3,033	3.49	129	4,749	2.72	55	5,435	1.01
Enterobacter aerogenes															
CRO	38	389	9.77	53	446	11.88	137	875	15.66	212	1,240	17.10	307	1,470	20.88
IPM	11	770	1.43	16	827	1.93	13	897	1.45	17	994	1.71	9	1,042	0.86
CIP	154	861	17.89	215	995	21.61	249	1,340	18.58	184	1,833	10.04	165	1,731	9.53
AN	38	570	6.67	52	733	7.09	63	1,216	5.18	44	1,594	2.76	44	1,823	2.41
Enterobacter cloacae															
CRO	187	861	21.72	158	768	20.57	404	1,343	30.08	660	1,887	34.98	1,305	2,746	47.52
IPM	12	1,805	0.66	14	1,869	0.75	20	2,025	0.99	21	2,460	0.85	43	3,163	1.36
CIP	681	1,915	35.56	577	1,943	29.70	734	2,367	31.01	1,146	3,319	3,4.53	1,279	3,853	33.19
AN	69	1,164	5.93	37	1,319	2.81	55	1,896	2.90	104	2,670	3.90	176	3,780	4.66
Klebsiella pneumoniae															
CRO	1,178	4,400	26.77	1,605	5,469	29.35	2,377	8,900	26.71	3,000	12,122	24.75	3,413	14,413	23.68
CIP	1,815	5,289	34.32	2,117	6,208	34.10	2,847	8,819	32.28	3,896	13,426	29.02	2,751	12,099	22.74
IPM	66	4,516	1.46	94	4,901	1.92	108	5,545	1.95	98	7,067	1.39	173	7,223	2.40
AN	363	3,963	9.16	380	4,935	7.70	470	8,440	5.57	579	12,156	4.76	448	13,804	3.25
Klebsiella oxytoca															

CRO	75	731	10.26	88	861	10.22	123	1,337	9.20	163	1,770	9.21	248	2,138	11.60
CIP	186	953	19.52	110	1,072	10.26	177	1,458	12.14	202	2,113	9.56	172	1,970	8.73
IPM	1	864	0.12	2	897	0.22	6	980	0.61	6	1,196	0.50	10	1,205	0.83
AN	63	651	9.68	46	780	5.90	70	1,247	5.61	66	1,825	3.62	77	2,055	3.75
Acinetobacter baumannii															
CAZ	150	218	68.81	131	193	67.88	57	174	32.76	46	178	25.84	30	141	21.28
CIP	150	217	69.12	110	197	55.84	50	148	33.78	21	148	14.19	25	147	17.01
IPM	66	229	28.82	45	199	22.61	23	189	12.17	21	196	10.71	23	185	12.43
Pseudomonas aeruginosa															
CAZ	797	5,688	14.01	862	6,115	14.10	816	6,366	12.82	1,062	7,527	14.11	837	7,562	11.07
CIP	1,041	4,038	25.78	1,089	4,490	24.25	1,034	5,134	20.10	1,231	6,464	19.04	1,292	7,005	18.44
IPM	1,299	5,684	22.85	1,483	6,165	24.06	1,212	6,631	18.28	1,460	7,748	18.84	1,363	8,150	16.72
Serratia marcescens															
CRO	17	504	3.37	46	618	7.44	23	671	3.43	43	857	5.02	53	949	5.58
CIP	32	602	5.32	45	719	6.26	60	753	7.97	55	1,035	5.31	70	1,090	6.42
IPM	2	495	0.40	3	595	0.50	1	562	0.17	0	700	0	2	672	0.29
AN	9	334	2.69	17	469	3.62	23	485	4.74	121	770	15.71	443	1,005	44.08

Morganella morganii															
CRO	29	590	4.92	30	609	4.93	69	895	7.71	65	1,097	5.93	75	1,223	6.13
CIP	104	643	16.17	151	683	22.11	220	949	23.18	229	1,270	18.03	174	1,207	14.42
IPM	26	570	4.56	53	559	9.48	56	609	9.20	36	633	5.69	60	589	10.19
AN	8	385	2.08	10	471	2.12	34	778	4.37	42	1,073	3.91	9	1,193	0.75
Enterococcus faecalis															
AMX	13	3,127	0.42	12	2,488	0.48	13	4,818	0.27	19	7,221	0.26	21	7,648	0.27
GM	356	2,774	12.83	454	3,437	13.21	2,053	6,036	34.01	3,972	7,848	50.61	4,215	6,446	65.39
VA	2	2,784	0.07	1	3,442	0.03	13	6,078	0.21	7	8,879	0.08	11	9,147	0.12
Enterococcus faecium															
AMX	373	463	80.56	336	406	82.76	508	633	80.25	881	1,071	82.26	822	991	82.95
GM	238	462	51.52	301	607	49.59	325	690	47.10	475	923	51.46	479	751	63.78
VA	18	465	3.87	23	316	3.73	22	707	3.11	24	1,015	2.36	8	950	0.84
Staphylococcus aureus															
OX	897	6,363	14.10	1,360	7,739	17.57	2,164	11,352	19.06	2,127	13,135	16.19	2,251	14,612	15.41
SXT	66	6,261	1.05	99	7,345	1.35	141	10,635	1.33	105	12,452	0.84	143	13,204	1.08
VA	1	6,242	0.02	0	7,336	0	1	8,925	0.01	1	9,543	0.01	2	9,817	0.02

Staphylococcus epidermidis															
OX	2,022	2,997	67.47	2,426	3,646	66.54	3,045	4,716	64.57	3,514	5,648	62.22	3,825	6,070	63.01
SXT	1,129	3,010	37.51	1,413	3,649	38.72	1,729	4,747	36.42	1,926	5,652	34.08	1,995	5,879	33.93
VA	11	3,005	0.37	7	3,650	0.19	6	4,339	0.14	6	4,798	0.13	2	4,890	0.04
Streptococcus agalactiae															
OX	0	1,547	0	0	1,958	0	0	3,246	0	0	4,269	0	0	3,948	0
SXT	8	426	1.88	2	535	0.37	155	1,307	11.86	9	2,336	0.39	10	1,406	0.71
VA	1	982	0.10	0	1,267	0	0	2,625	0	1	3,469	0.03	0	3,381	0

AMX: Amoxicillin; AMC: Amoxicillin-clavulanic acid; AN: Amikacin; CRO: Ceftriaxone; CAZ: Ceftazidime; CIP: Ciprofloxacin; GM: Gentamicin; IPM: Imipenem; OX: Oxacillin; SXT: Cotrimoxazole; VA: Vancomycin

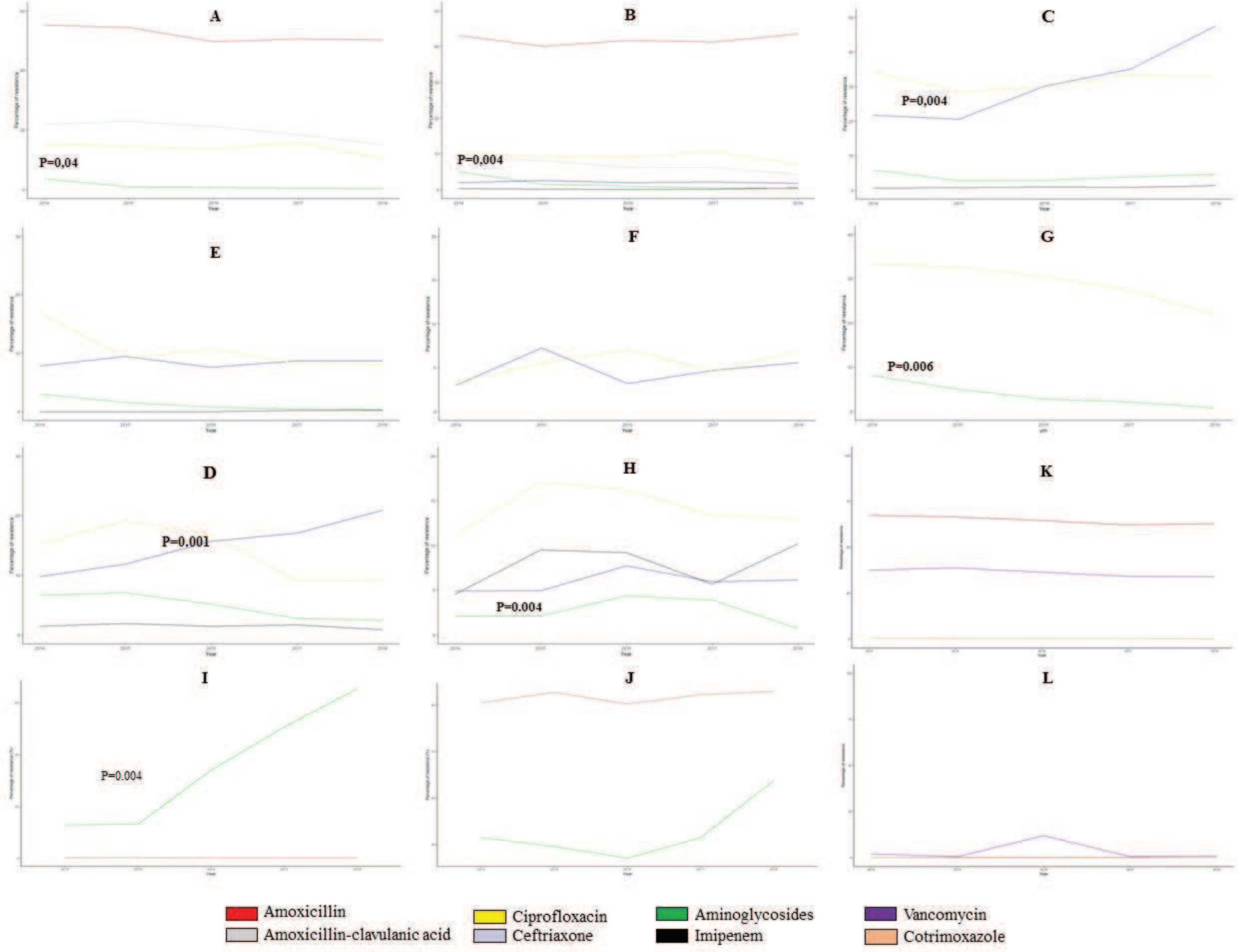

Figure S1. Evolution of resistance percentage of key antibiotics in bacterial species isolated by the different laboratories included in our study from January 2014 to December 2018. **A.** *E. coli;* **B**. *P. mirabilis*; **C.** *E. cloacae*; **D.** *E. aerogenes*; **E.** *K. oxytoca*; **F.** *S. marcescens*; **G.** *K. pneumoniae;* ***H.*** *M. morganii;* **I**. *E. faecalis;* **J.** *E. faecium;* **K.** *S. epidermidis;* **L.** *S. agalactiae*

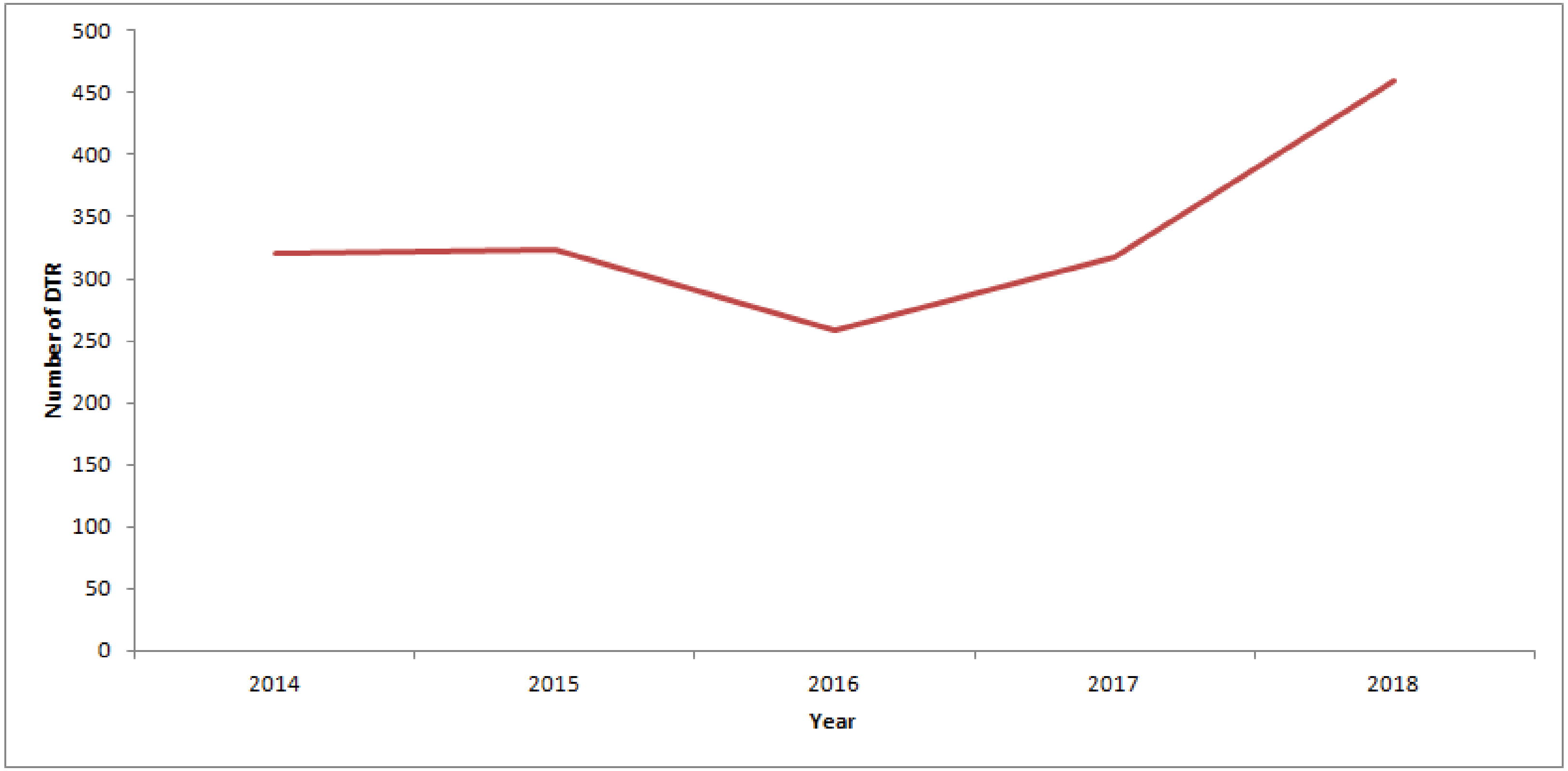

Figure S2. Evolution of the number of strains carrying a DTR phenotype between 2014 and 2018.

Figure S2.
